# RB49-like Bacteriophages Recognize O Antigens as One of the Alternative Primary Receptors

**DOI:** 10.3390/ijms231911329

**Published:** 2022-09-26

**Authors:** Alexandr D. Efimov, Alla K. Golomidova, Eugene E. Kulikov, Ilya S. Belalov, Pavel A. Ivanov, Andrey V. Letarov

**Affiliations:** 1 Laboratory of Microbial Viruses, Winogradsky Institute of Microbiology RC Biotechnology RAS, 117312 Moscow, Russia; 2 Faculty of Biology, Lomonosov Moscow State University, 119991 Moscow, Russia

**Keywords:** enterobacteria, O antigen, RB49-like bacteriophages, phage-host recognition, phage receptors

## Abstract

The power of most of the enterobacterial O antigen types to provide robust protection against direct recognition of the cell surface by bacteriophage receptor-recognition proteins (RBP) has been recently recognized. The bacteriophages infecting O antigen producing strains of *E. coli* employ various strategies to tackle this nonspecific protection. T-even related phages, including RB49-like viruses, often have wide host ranges, being considered good candidates for use in phage therapy. However, the mechanisms by which these phages overcome the O antigen barrier remain unknown. We demonstrate here that RB49 and related phages Cognac49 and Whisky49 directly use certain types of O antigen as their primary receptors recognized by the virus long tail fibers (LTF) RBP gp38, so the O antigen becomes an attractant instead of an obstacle. Simultaneously to recognize multiple O antigen types, LTFs of each of these phages can bind to additional receptors, such as OmpA protein, enabling them to infect some rough strains of *E. coli*. We speculate that the mechanical force of the deployment of the short tail fibers (STF) triggered by the LTF binding to the O antigen or underneath of it, allows the receptor binding domains of STF to break through the O polysaccharide layer.

## 1. Introduction

The recognition of the host cell surface by a bacteriophage particle is critical for successful infection. Adsorption immunity, where the phage is unable to attach to the host cell lacking a suitable receptor [1,2,3], determines a larger part of bacterial phage resistance. Two distinct steps are required for a successful infection by tailed bacteriophage: (i) reversible phage adsorption to the primary receptor(s) on the bacterial cell surface, followed by (ii) irreversible adsorption often requiring recognition of the final (secondary) receptor. The latter interaction triggers structural rearrangements of the virion, resulting in the ejection of the viral DNA. In some viruses the recognition of the primary receptor is prerequisite for correct interaction with secondary receptor(s). Such a strategy is frequently found in podoviruses [4,5,6,7]. An alternative strategy relies on independent phage interactions with the primary and secondary receptors [3,7]. The phage binds its primary receptor with no structural changes of virion, anchoring the viral particle to the potential host cell and giving it an opportunity to reach the secondary receptor. If the secondary receptor is directly exposed on the cell surface, some bacteriophages may bypass the interaction with the primary receptor(s) for the infection. In the viruses adopting this strategy, the recognition of the primary receptors may increase virus adsorption rate [8] and increase the time frame available for successful penetration of the phage receptor binding protein (RBP) through the bacterial surface structures, masking secondary receptors [9,10]. The latter function appears to be essential in many phages of the Gram-negative hosts, where O antigen efficiently shields the cells against phages, not possessing O antigen-specific adhesins/depolymerases (reviewed in [3]). Therefore, the O antigen is one of the major determinants of the actual cell sensitivity to bacteriophages [7,11,12,13], either making for a protective layer masking the cell surface or serving as a tag marking the susceptible host cell for phage docking. 

In a recent large study of coliphage interactions with host cells [14], two groups of broad-host range myoviruses were identified. Vequintavirinae family phages featuring complex adsorption apparatus with multiple RBPs were found to recognize a highly conserved cell surface polysaccharide–enterobacterial common antigen (ECA); T-even-related phages had no means to recognize ECA but were also able to infect O antigen producing strains. T-even-related viruses are often isolated from various natural environments [15,16,17,18,19] and predominate among the isolates featuring a relatively wide host range in respect of Enterobacteriaceae strains [15].

The host cell recognition by most myoviruses falls in between two of the aforementioned strategies. Many myoviruses specifically recognize their primary receptor using the virion molecular devices called long tail fibers (LTFs). The LTF binding to the cell surface triggers the phage baseplate rearrangement [20,21,22,23], initiating tail contraction. Once the baseplate rearrangement and tail contraction have started, the structural changes of virion become irreversible, leading to either successful infection or virion inactivation. Thus, the interaction with the primary receptor may lead (though, not always immediately) to the irreversible phage adsorption or, if something goes wrong, to the wasting of the viral particle. In most of the myoviruses, recognition of the secondary receptors becomes available only after the baseplate rearrangement [20,23]. In T-even-like bacteriophages, the secondary receptor is recognized by the short tail fibers (STF) folded under the baseplate before infection, being deployed only after LTF binding to their receptors [20,24,25]. The STFs binding to the secondary receptor tightly attaches the phage particle to the cell surface, damping the force of the tail contraction and preventing phage expulsion from the cell surface. However, in phage P1, LTF binding appears to be sufficient to hold the virion at the cell surface; although, P1 has a much longer tail allowing the tail tube to cross the gap between the baseplate and cell surface, formed due to extension of the flexible LTFs by repulsion of the virion from the cell surface by the force of the tail contraction [20].

The sequence of events during host infection is best studied for the bacteriophage T4 [20,24]. The similarity of most of the structural proteins strongly suggests that the general pathway of the infection is essentially the same for other T-even phages (such as T2 and T6) and more distantly related Pseudo-T-even and Schizo-T-even viruses [26], re-classified now within the family Straboviridae, genera *Krishvirus*, *Mylasvirus*, *Pseudotevenvirus*, *Schizotequatrovirus*, *Slopekvirus* and *Tulanevirus* (https://talk.ictvonline.org/taxonomy/, accessed on 21 July 2022). 

RB49 is a pseudo-T-even bacteriophage with virion morphology very similar to the phage T4 [27,28]. Most of its structural proteins are related to the cognate T4 proteins. However, the structural basis of the receptor recognition by the LTFs is different in RB49. In T4, the distal part of the distal half of the LTF is built by a trimeric gp37 protein, C-terminal region of which forms a thin needle ended with the receptor-binding domain [25]. In RB49, as well as in the vast majority of the T-even-like bacteriophages, gp37 carries a C-terminal self-cleaving chaperone domain [29,30]. After removal of this domain, a monomer of gp38 adhesin is attached to each of six LTFs. The C-terminal domain of gp38 is responsible for the receptor recognition. The structure of the complex of gp37 C-terminal fragment and gp38 was solved for the *Salmonella enterica* phage S16 [31]. This protein is homologous to RB49 gp37, so its structural organization is predicted to be very similar [32].

RB49 was originally isolated on the rough *E. coli* B strain. It also infects *E. coli* K-12 strain and its derivatives, though somewhat less efficiently [29]. Therefore, RB49 should recognize some structure(s) at the *E. coli* outer membrane (OM) surface as its primary receptor(s). In some host strains this is OmpA protein [29], while other strains could employ large conservative OM transporter proteins or lipopolysaccharide (LPS) core oligosaccharide (core-OS). Interestingly, some phages closely related to RB49 can efficiently infect the O antigen producing host strains in which the OM surface is protected from direct interaction with phage RBPs [7,33]. Here, we present evidence that some of the phage-host systems, the O polysaccharide (OPS), serves as the primary receptor. 

## 2. Results

### 2.1. RB49-like Bacteriophages Have Different Adsorption Specificity towards E. Coli Strains with Different O Antigen Types

The bacteriophage RB49 was originally isolated on *E. coli* B [28] and is able to infect *E. coli* K12 (rough mutant of an O16 parental strain [33,34]) and K12 derivatives such as *E. coli* C600, so all these original strains missed the O antigen. Bacteriophages Whisky49, Cognac49 and Brandy49 that are closely related to RB49 [33] were isolated on O antigen producing *E. coli* strains F17 (new serotype), F5 (O28) and 4s (O22), respectively. We cross-tested these phages against all the above-mentioned strains and their rough derivatives (see Materials and methods section for detail) (Table 1). All four bacteriophages formed plaques on the host strain F5, showing on it the maximal biological titer values, though we considered this strain as the optimal host and determined the efficiency of plating (EOP) with respect to the plating on the F5 strain.

Only Brandy49 was able to infect the 4s host strain or its rough derivative. At the same time, phages Brandy49 and Whisky49 were able to infect the strain F17, while RB49 and Cognac49 did not. Interestingly, all the phages were able to grow on the usual laboratory K-12, C600 and BL21 strains (though Whisky49 showed slightly reduced EOP on these hosts). At the same time, the rough derivative of the F17 strain was resistant to all these viruses. The rough derivative of the F5 strain remained sensitive to all the phages, but Whisky49 showed reduced EOP on it with the level of reduction similar to that observed on the laboratory rough *E. coli* strains. These results indicate that, despite their known potential of cell surface shielding, O antigens may facilitate the infection by RB49-like phages on some host strains. 

### 2.2. Genetic Determination of the Host Range

In T-even related phages recognition of the cell by phage LTFs mediates phage attachment and triggers the tail contraction (reviewed in [24]). The observed inability of the phage to get adsorbed by the host cell without extensive phage decay upon contact with the phage-resistant host cells can be explained by phage LTFs not binding their counterparts on the cell surface, but not by the inability of the STF to bind to their cognate receptors. 

**Figure 1:**
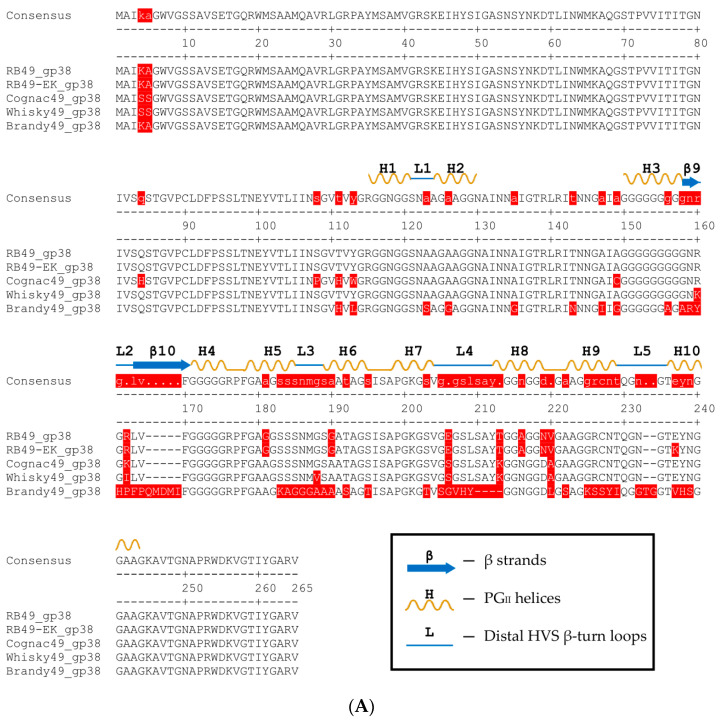

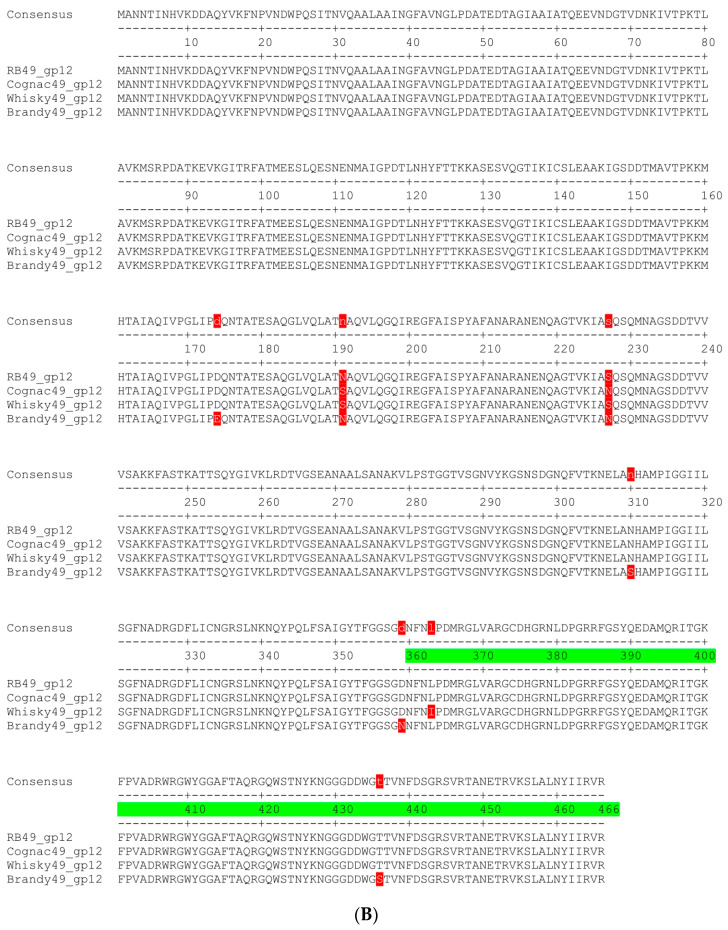
Comparison of the receptor-recognition proteins sequences. Mismatches in the sequence colored red. (**A**) The receptor-binding protein of the LTF gp38. The secondary structure elements of the C-terminal receptor binding domain are indicated above the consensus sequence in accordance with their numbering in [31]. (**B**) The short tail fiber protein gp12. Putative receptor-recognition domain colored green.

To assess the structural basis for our hypothesis, we compared the protein sequences of the LTF adhesin gp38 (Figure 1A) and STF protein gp12 from all the phages under the study (Figure 1B). As was expected, the sequences of gp12 were almost identical. Only three a.a. changes were observed in the receptor-recognition domain (corresponding in T4 phage to the residues 396–527 [35]). Only one mutation L363I was separating Whisky49 gp12 sequence from the RB49 and Cognac49 proteins.

At the same time, the protein sequences of gp38 of these phages shared less of their identity. The proteins from RB49, Gognac49 and Whisky 49 have multiple amino acid polymorphisms, but only eight a.a. positions within the predicted receptor-recognition domain were specific to Whisky49, potentially explaining its ability to infect *E. coli* F17 strain, resistant to both RB49 and Cognac49 (Table 1). The gp38 from phage Brandy49 was much more divergent from the other three viruses that were associated with the broadest host range.

**Figure 2:**
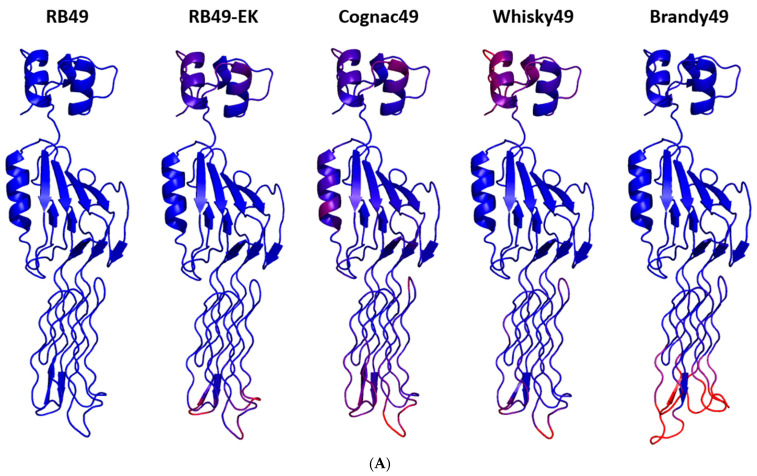

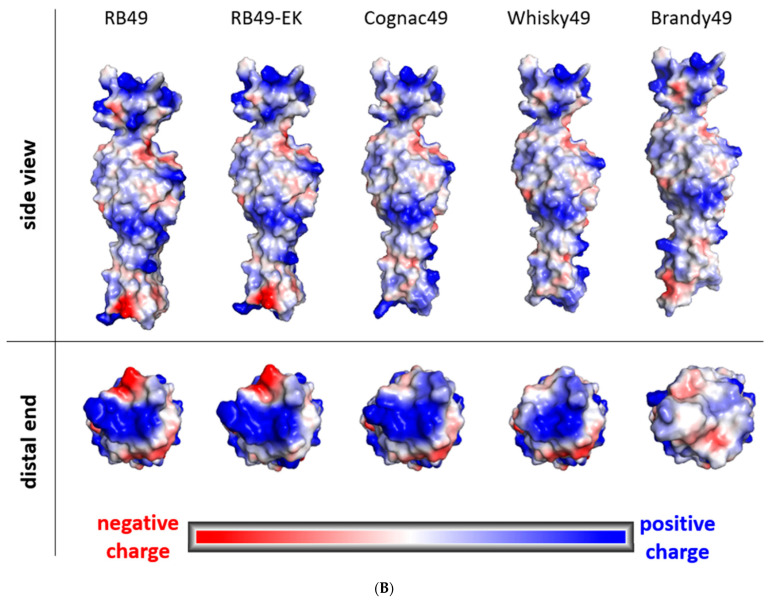
AlphaFold modelling of the structures of gp38 from RB49-like bacteriophages. (**A**) Backbone view. (**B**) Surface view. The color scale reflects the surface electric charge.

The AlphaFold modelling of the gp38 structures predicted only a moderate difference in the backbone chain conformation between the proteins from different phages (Figure 2). At the same time, the shape and charge of the surface formed by the loops L2 and L4 (according to [31]; see Figure 1A) on the distal (in respect to the phage baseplate) end of the molecule were significantly different in Brandy49 compared to the other bacteriophages (Figure 2B). The genetic evidence suggests that these loops are involved in the formation of the receptor-recognition center (refs. [29,31] and this work) defining the bacteriophage host range. In Brandy49 phage, the area of the putative receptor-binding site is larger but it is almost depleted of charged amino acids, while in other phages most of the surface in this area is positively charged (Figure 2B). The decreased positive charge density in gp38 of the phage Brandy49 may reduce its ability to form salt bridges with a charged or highly polar receptor molecule such as polysaccharides or negatively charged loops of the host outer membrane proteins.

We concluded that the differences in the recognition of the F17 strain are due to different binding specificities of the LTF adhesin gp38. Brandy49 had the most divergent gp38 sequence (compared to the consensus) that was associated with its broadest host range.

Since the sequence of the phage RB49 gp38 is very similar to Whisky49 and Cognac49, it should be possible to obtain an RB49 mutant able to grow on *E. coli* F17 host. We plated three independently grown high titer RB49 stocks onto the strain F17 lawns (ca. 10^9^ PFU per plate). In two cases we obtained several plaques. Two mutant phage isolates (one per original stock) were purified and sequenced. The genomic sequences obtained were identical to the original RB49 sequence, except for point mutations leading to the a.a. substitutions in gp38: E230K in one case and E230K and Y231S in the other. Interestingly, these a.a. positions are identical among the natural gp38 sequences of all three phages (Figure 1A), so there is more than one way for RB49-like gp38 to attain the specificity towards the *E. coli* F17 surface. This RB49 derivative, named RB49-EK, was added to the phage set (Table 1).

### 2.3. Sensitivity of the Host Strains Rough Mutants to RB49-like Phages

All the phages under the study were able to grow on the laboratory rough strains C600, K-12 MG1665 and BL21. This means that their LTFs can recognize some receptor(s) on the immediate cell OM surface. It has been previously reported that OmpA protein may serve as a receptor for the phage RB49 [29]. We confirmed that the Keio collection [36] OmpA knockout strain is fully resistant to RB49. This strain was also resistant to all the phages included in the present study, but not to Brandy49. The complementation of the OmpA from the plasmid restored the sensitivity of the mutant to all the phages, confirming that OmpA deletion, but not any other unexpected genetic difference(s), was responsible for the resistance. 

At the same time, these phages were able to infect the O antigen producing strains (F17 and F5), the O antigens of which have been previously shown to be very effective in the protection of the host cell outer membrane (OM) receptors from the interaction with bacteriophage RBPs [9,11,37] (though the O28 O antigen of F5 strain was slightly less protective than the other two, it reduced the phage infection by several orders of magnitude [9]).

The infectivity of these phages against the O antigen producing strains may be explained by two alternative mechanisms: (i) the LTF tip is able to somehow penetrate through the O antigen and to bind its final receptor or (ii) LTFs can recognize some O antigen types as an alternative receptor, displaying double or even multiple O antigen-mediated specificity. 

To assess the hypothesis applicable to our case, we tested the RB49-like phage set against rough derivatives of the *E. coli* strains F5 and F17 (Table 1). All the phages, except Brandy49, were unable to infect the rough mutant of the F17 strain while the rough derivative of F5 remained sensitive to all the phages, though the EOP of Whisky49 dropped as it was seen on other rough host strains. We then complemented F17 Δ*wbb*L mutant using the pWbbL plasmid [37]. In the complemented strain, the sensitivity to Whisky49 and RB49-EK phages was restored (Table 1), as well as the ability to produce the O antigen [37]. This indicates that on the strains F17 and F5 all the phages, except for Brandy49, may directly use O polysaccharide as the primary receptor, while on the rough *E. coli* K12 these viruses recognize OmpA.

Surprisingly, no phage-sensitive phenotype was restored when we introduced the pOmpA plasmid into the phage-resistant derivatives of the laboratory strains or into the phage-resistant rough derivatives of the strains F5, F17 or F17 *wbb*L. This may indicate that the phages recognize, not the OmpA protein itself, but some other outer membrane receptor, the surface expression of which depends on OmpA. Alternatively, one may suggest that incorporation of the plasmid-encoded OmpA into the OM may be hindered in the presence of a functional genomic copy of OmpA. However, further analysis of the receptor(s) recognized by these phages on the rough host strains was out of scope of this study.

### 2.4. O Antigen Alterations in Phage-Resistant Mutants

**Figure 3:**
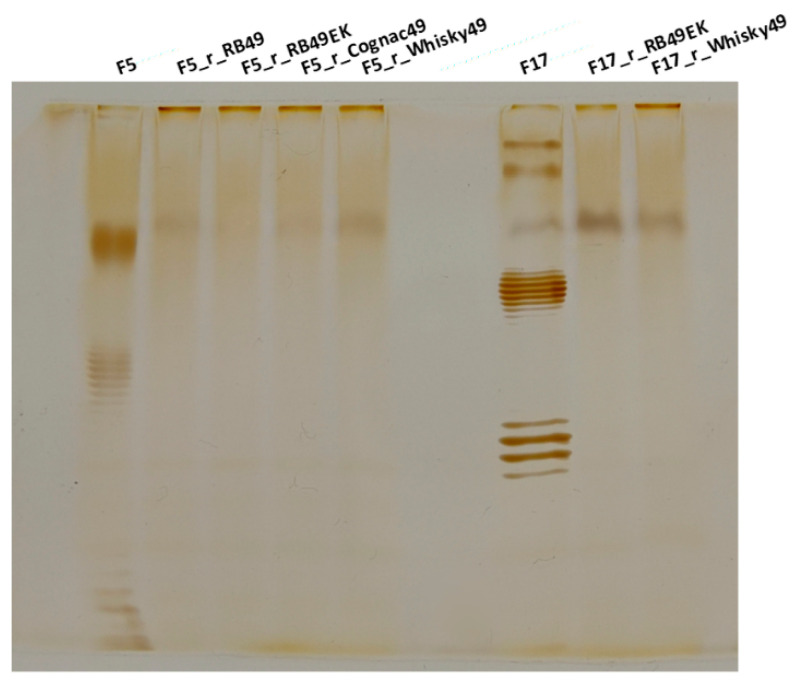
LPS profiles (SDS-PAGE) of the derivatives of the strains F5 and F17 selected for resistance to the phages RB49, RB49EK, Cognac49 and Whisky49.

The results described in the previous section suggest that, in some of the host strains, O antigen serves as a LTF receptor, at least for RB49, Whisky49 and Cognac49 phages. To further confirm this conclusion, we isolated *E. coli* F5 natural mutants resistant to each of these phages, and F17 strain mutants resistant to Whisky49 and RB49-EK phages. These mutants (three independent mutant clones were tested for each phage-host pair) demonstrated cross-resistance to all the phages (Table 2). We analyzed the LPS profiles of these strains using electrophoresis. The derivatives of the strains F5 and F17 were depleted of the O antigen synthesis (Figure 3) being rough mutants. This was further confirmed by the fact that these mutants gained sensitivity to the T5-like phage FimX (Table 2) that may be used as a probe to detect the reduced OM shielding by the O antigen [7,9,10].

### 2.5. Phage Brandy49 Has a Distinct Mechanism of O Antigen Penetration

Summarizing the results mentioned above, we conclude that the phages RB49, RB49-EK, Cognac49 and Whisky49 penetrate through the host O antigen of the strains F17 and F5 by means of the recognition of their O polysaccharides as primary receptors. However, the data indicate that the mechanism underlying the infectivity of phage Brandy49 against the O antigen producing strains is different (see below).

Phage Brandy49 isolated on *E. coli 4s* showed a much broader host range compared to the other four strains included in this study, clearly recognizing different primary receptor(s). Noteworthy, the ability of Brandy49 to infect the *E. coli* K-12 *ompA* mutant is in good agreement with the conclusion that OmpA protein is, indeed, an LTF receptor of RB49-like phages (except for Brandy49) under the study, since the sequence of the short tail fiber protein gp12 in Brandy49 is almost identical to the other phages (Figure 1B). To check if the receptor of Brandy49 is connected to LPS, we tested the infectivity of this phage on a set of the mutants deficient in O antigen and core oligosaccharide synthesis: 4sGTR—lacking O antigen additional glycosylation; 4sR (wclH)—lacking O antigen; 4s *waaG*—lacking the outer core and 4s *waaC*—a deep-rough mutant with only two Kdo residues remaining from the outer core (for detailed description of these mutants see [7]). All these mutants were fully sensitive to Brandy49. 

The mutants of all the strains selected for resistance to other phages were also sensitive to Brandy49 (Table 2). 

**Figure 4:**
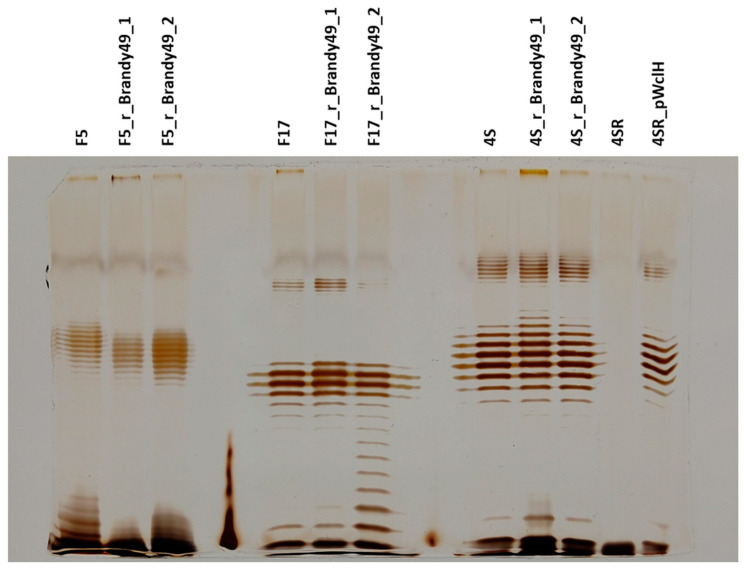
LPS profiles of the strains F5, F17 and 4s mutants, selected for the resistance to the phage Brandy49.

To test Brandy49 interactions with the host O antigen, we selected strains 4s, F17 and F5 for resistance to this phage and checked their O antigen production status. The mutants obtained remained smooth with the LPS patterns indistinguishable from the parental strains (Figure 4), so the O antigen itself was not used as the receptor by the Brandy49 phage. To further confirm this finding, we selected the strain 4sR for Brandy49 resistance and then complemented the obtained mutants with pWclH plasmid. We confirmed that the O antigen biosynthesis had been restored (Figure 4), nevertheless all the clones tested (*n* = 3) remained resistant to Brandy49. This result confirms that the O polysaccharide is not used as either a main or alternative LTF receptor during infection of these strains by the phage Brandy49.

## 3. Discussion

Bacteriophages are once again considered as perspective biocontrol agents for many applications, [38,39,40,41] including phage therapy (PT) that may provide at least an interim solution for the alleviation of the global crisis caused by the spread of multidrug-resistant bacterial pathogens [39,42,43,44,45]. Broad host range bacteriophages are highly demanded for PT. For some pathogens, such as Staphylococcus aureus, the bacteriophages active against the vast majority of the clinically relevant strains were described and used in commercial phage cocktails [46]. However, for many other pathogenic bacterial species broad host range phages were not found yet. 

The recognition of the host cell surface by the virion RBP (s) is a key step of the phage lytic life cycle that largely determines the bacteriophage host range [3,47]. Although different bacteriophages, including coliphages, recognize a great variety of the cell surface molecules as their receptors [3], the accessibility of the receptors present on the surface of the *E. coli* outer membrane is greatly reduced by their shielding from the layer of O antigen. In most of the O-serotypes, the O antigen protects the OM surface very efficiently, giving the cells complete resistance to many bacteriophages potentially able to infect these strains if the O antigen is removed, for example, due to mutation [7,9,10,48,49]. 

The bacteriophages use different strategies to penetrate through the O antigen layer. Some phages employ enzymatically active tail spikes [50,51], others use accessory adsorption devices specifically recognizing O antigen, and allowing (by some poorly understood mechanism(s)) the main RBP to penetrate to the secondary receptor on the OM surface [10], see also [3] for review). Any strategy requires specific recognition and binding of the O antigen by the bacteriophage RBPs. *E. coli* is an extremely divergent bacterial species with more than 200 O-serotypes differing by the structure of the O-polysaccharide repetitive unit described [9,52,53]. Many of these serotypes may feature pathogenic potential and cause different infections in humans and in animals [54,55,56,57,58]. For example, more than 50% of the urological infections in patients without anatomical anomalies are caused by uropathogenic *E. coli*, represented by more than 15 serotypes [59]. Such a degree of natural molecular diversity on a “cheap” basis of polysaccharide combinatorial chemistry makes *E. coli* a very difficult object for the formulation of bacteriophage cocktails for therapy. The empirical search for broad host range phage isolates is a keystone of PT cocktails development by the Microgen company (Moscow, Russia)—the leading Russian commercial producer of phage-based drugs, though until recently these phages were not characterized in any detail [60,61]. Surprisingly, sampling a commercial phage mixture against *E. coli* strains featuring efficient non-specific OM surface protection by the O antigen yielded three closely related isolates of RB49-like bacteriophages Cognac49, Whisky49 and Brandy49 [33]. This result was counter-intuitive, because RB49-like phages were never found to carry multiple RBPs for the recognition of the alternative primary receptors. The receptor recognition domain of gp38 of a RB49-like phage is relatively small (about 180 a.a.) and, therefore, is unlikely to be accommodated in its structure multiple receptor binding sites. On the other hand, all the isolates obtained and the original RB49 phage are able to grow on some rough *E. coli* strains, such as C600, which means that their gp38 should recognize some molecules located on the OM surface, shielded by the O antigen in the non-rough *E. coli* strains used for the isolation of the phages used in this study. Moreover, each of these phages can infect multiple host strains producing structurally distinct O-polysaccharide molecules [7,9,48,49]. 

Our results suggest that on the strains F5 and F17 all the phages use the O antigen as the primary receptors by the phages RB49, Cognac49 and Whisky49 but not by Brandy49. In the strain F17, the O antigen appears to be the only possible LTF receptor since the selected phage-resistant mutants turn rough, and the artificially created rough derivative (*wbb*L knockout) of this strain is resistant to all four phages. In the F5 strain, both the O antigen and other unidentified OM-surface structure can be used as alternative receptors. The alternative explanation that, in the strain F5 the O antigen surface expression is dependent on some other structure serving as the receptor for the bacteriophages, cannot be excluded, but it appears less likely based on the current knowledge of LPS biosynthesis.

Bacteriophage Brandy49 that was able to infect all the O antigen-producing strains tested in this study does not bind the O antigen (at least on the strains 4s, F17 and F5). Moreover, the restoration of the O antigen biosynthesis in rough variants of the strains 4s and F17 pre-selected for Brandy49 resistance did not return the sensitivity to the phage. This fact indicates that Brandy49 uses some receptor on the OM surface that is not structurally or functionally linked to O antigen expression and the binding of this unidentified receptor cannot be replaced by the interaction with the O polysaccharides. Given the fact that the O antigens of the strains 4s and F17 were shown to effectively shield the cell surface [7,37,49] we can conclude that the LTFs of Brandy49 have an unusual ability to penetrate through the O antigen layers of different structural types to bring the receptor-recognizing moiety of gp38 into contact with the OM surface. The molecular mechanism of such ability remains elusive. However, the decreased charge density of the “bottom” surface of the receptor recognition domain (as predicted by AlphaFold modelling, [62]) may contribute to the LTF tip penetrability, preventing unnecessary interactions with highly polar polysaccharide molecules. Interestingly, the proposed ability of the LTF tip made of gp38 protein to penetrate through the O antigen to reach the conserved receptor(s) at the OM surface appears hardly compatible with the observations by Hu [20] and Fokine [63]. These researchers demonstrated, using cryo-electron microscopy approaches, that in the phage T4 related to RB49-like viruses (though more distantly) the LTFs are folded upwards and interact with the tail shaft and with the capsid until the interaction with the host cell. T-even-like phage particles in the moment of its initial collision with the host cell represents a bulky object with no needle-like LTFs protruding outwards. Therefore, the O antigen should be even more effective in protecting the cell from T-even-like phage infection compared, for example, with T5-like viruses, the main RPB of which, pb5, is located at the tip of the central tail fiber [64,65]. As we have demonstrated previously, T5-like phages are completely adsorption-restricted by the O antigens of the strains 4s, UP1 and F17 used in this study, and almost completely restricted by F5 strain O antigen [7,9,48,49]. This may indicate that the LTF pre-adsorption conformation in RB49-like phages may differ from phage T4. In phage T4, the fixation of the LTFs in the “up” position is in part dependent on the interaction of the LTF with the collar and whiskers formed by the Wac protein [66,67,68]. Noteworthy, the Wac protein of RB49-like phages caries the C-terminal moiety unrelated to that T4 Wac [66,67].

**Figure 5:**
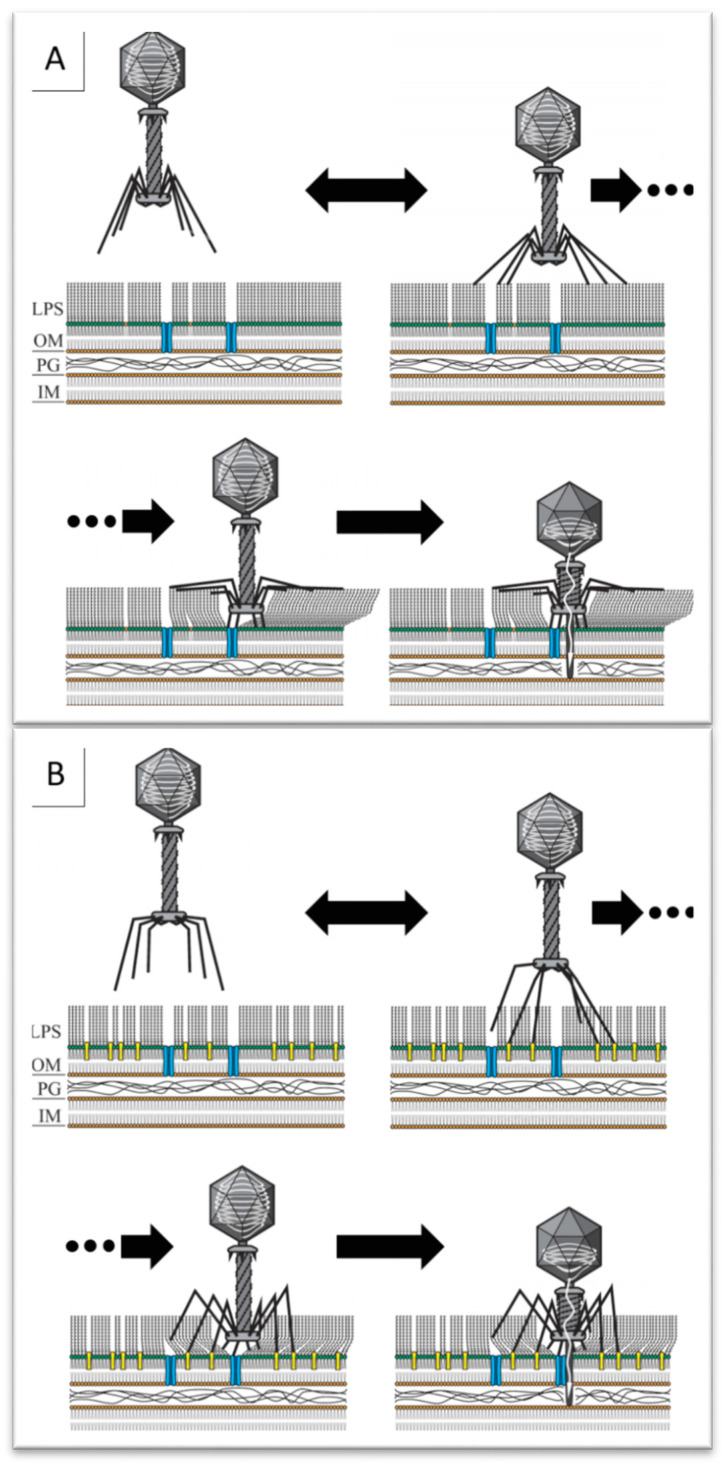
Model of the O antigen-producing host infection by RB49-like phages. (**A**) The LTFs of the phages RB49, Cognac49 or Whisky49 recognize O-polysaccharides of some strains (such as F5 or F17) triggering the baseplate rearrangement. The mechanical force of the deploying STF allows their C-terminal receptor binding domains to get through the O antigen layer to the receptors at the OM surface. (**B**) The LTFs of the phage Brandy49 are able to penetrate through the O antigen layer without binding to the OPS to recognize their receptors at the OM surface.

The interaction of the LTFs with the outermost O-units of the O antigen chains (that should only be accessible to the bulky virion) is likely to trigger the phage baseplate rearrangement, while the O antigen layer still separates the baseplate and the OM surface. We may hypothesize that the mechanical force of the deployment of the phage STF (gp12) from the bottom of the baseplate [20,25] may force the receptor-binding heads of the gp12 trimers through the O-polysaccharide layer (Figure 5). In the single particle cryo-tomography reconstructions, the phage T4 virion, bound to the host surface, appears “standing” on six extended STFs and the distance between the baseplate and the OM surface is about 20 nm which is comparable to the estimated O antigen thickness in the *E. coli* strains able to produce this structure.

Many of the prototypical bacteriophages, such as RB49, were originally isolated on laboratory bacterial strains, most of which are rough mutants. This circumstance casts a serious shadow on the study of bacteriophages isolated on these “domesticated” bacteria. The same applies to bacteriophage cultivation on the attenuated bacterial strains, ultimately producing phages missing their original biological features and abilities due to the relieved pressure of natural selection. Fully understanding the details of phage biology is important for both applied and basic science. We need to shift our research paradigm and widen our scope, considering all aspects of phage biology relevant to the phage cycle, such as primary/secondary host recognition mechanisms, etc.

The ability of relatively small LTF adhesin, such as gp38, to recognize multiple structurally different types of the OPS is counter-intuitive and merits fine structural/biochemical studies. Approaches such as the determination of the structure of the gp38—OPS complexes and in vitro modelling of these interactions using purified O antigens or OPS-decorated nanoparticles may be instrumental.

## 4. Materials and Methods

### 4.1. Bacterial and Bacteriophage Strains 

All the bacterial strains used were from our laboratory collection. *E. coli* strains 4s, F17 and F5 were previously isolated from horse feces [9,48,69]. Strain 4s produces O22-like O antigen [49]. Its mutants 4sI—lacking O antigen O-acetylation (wclK-); 4s-GTR—lacking O antigen lateral glycosylation; 4sR—rough mutant depleted of O-unit synthesis (wclH-; 4sG—lacking the outer core oligosaccharide (waaG-); 4sC—having the core-OS reduced to two Kdo residues (waaC-) were described in [7,49]. Strain F5 produces O28 type O antigens, and strain F17 belongs to a novel O antigen serotype [48].

Bacteriophage RB49 was from our laboratory collection. The RB49-like bacteriophages Cognac49 (GenBank ID: MZ504877), Whisky49 (MZ504878) and Brandy49 (MZ504876) were isolated from commercial therapeutical phage mixture produced by Microgen company, Russia [33]. Bacteriophage RB49 (NC_005066.1) was from the collection of our laboratory.

Phage FimX is a mutant of the phage DT571/2, closely related to the phage T5. FimX is depleted of the lateral tail fibers and, therefore, is able to infect only rough strains of *E. coli* or the strains in which the non-specific outer membrane shielding by the O antigen is noneffective [7,9,10]. 

### 4.2. Bacteriophage Adsorption Assay

The mid-log phase liquid culture of appropriate host strain (OD_600_ = 0.6) grown in LB medium was mixed with diluted phage to obtain the final phage concentration of about 4 × 10^3^ p.f.u. mL^−1^. The mixture was incubated for 10 min at 37 °C. Then, the mixture was centrifuged at 12,000× *g* for 3 min to pellet down the bacterial cells. After that, 50 μL of the supernatant was plated onto the *E. coli* C600 lawn by the conventional double-layer technique to count the non-adsorbed phage. To estimate the initial phage counts, the same dilution of the phage was made in LB medium without cells and processed in the same way. The experiment was performed in triplicate.

### 4.3. Selection of Bacteriophage-Resistant Mutants

The phage-resistant mutants were selected by plating the bacterial culture to the plates containing the phage agar (ca. 10^8^ p.f.u. per plate) as previously described [12].

### 4.4. Genomic Sequencing

Phage DNA was extracted as described earlier [33]; a concentrated phage stock was treated with DNAse and RNAse and the DNA was extracted using CTAB [70]. The genomic sequencing was performed with a IonTorrent sequencer with standard chemistry. The contigs were assembled with SPAdes [71]. 

### 4.5. Lipopolysaccharide (LPS) Profiling 

SDS-PAGE profiling of the LPS of bacterial strains was performed as described in [7]. Briefly, the bacterial biomass collected from the plate was resuspended in the standard Laemmli protein PAGE loading denaturing buffer containing sodium dodecyl sulfate (SDS) and digested with proteinase K. The samples were then run on a conventional protein SDS-polyacrylamide electrophoresis. LPS complexes with SDS were resolved in 12% PAGE by their molecular mass, and polysaccharide part of LPS was oxidized in gel using periodic acid generated in situ from sodium periodate and acetic acid, yielding numerous aldehyde moieties connected to polysaccharide links, making sugar residues coupled by glycoside bonds reducing, i.e., reactive towards silver-ammonia complex ions. The gels were developed using citric acid-formaldehyde reducing reagent according to the protocol [7]. 

### 4.6. Construction of pOmpA Plasmid and Complementation 

Gene *ompA* was amplified from K12 MG1665 strain by PCR with the primers OmpA_F (ACTTTACATCGCCAGGGGTG) and OmpA_R (TCGCATGAAGCAAACCATTC). The PCR product was cloned into a pGEM-T vector (Promega, Madison, WI, USA) according to the manufacturer’s recommendations. The clone with the insertion orientation under the control of lac promoter was selected by PCR screening with the primers M13R (Fermentas) and OmpA_R. The mutant cells to be complemented were transformed using this plasmid. For the complementation experiments, the plates were supplemented with 0.1 mM of IPTG inducer.

The complementation of the strains F17 wbbL and 4sR (wclH-) was performed in the same conditions using the plasmids previously described [48,49].

### 4.7. Bioinformatic Analysis and Structural Modelling of Phage Proteins

The protein sequences of the proteins of interest were extracted from the annotated genome sequences listed in the Section 4.1. The multiple protein sequence alignments were made using the online tool MAFFT (https://mafft.cbrc.jp/alignment/server/, accessed on 10 July 2022) with further manual curation.

Protein structure prediction was performed using AlphaFold2 (EMBL-EBI, Hinxton, UK). Amino acid sequence of the gp38 protein of each phage under this study was loaded as a monomer into a form on the website “https://colab.research.google.com/github/sokrypton/ColabFold/blob/main/AlphaFold2.ipynb (accessed on 12 May 2022)” with the default settings. The analysis of the obtained structures was performed in PyMOL. Msd structure coloring was applied with alignment relative to RB49. Blue-colored structures are similar, red-colored structures are dissimilar, with intercolor according to similarity level. Vacuum electrostatics surface imaging modeled in PyMol graduated from red (−) to blue (+).

Studies involved no animals or humans, so no ethical approval was required.

## 5. Conclusions

The O antigen of enterobacteria was recently recognized as one of the major factors shaping the host ranges of bacteriophages infecting these organisms. The paradigm of the non-specific O antigen shield is also likely to be relevant for many other groups of Gram-negative bacteria. Our results reveal an unusual strategy of some T-even-related bacteriophages to infect the host strains producing different O antigen types. Instead of using O-polysaccharide depolymerases or recognizing conserved surface-exposed molecules, such as ECA, NFR or bacterial cellulose, these viruses attach directly to the OPS. Our results suggest the possible adaptive value of the long-known structural “design” of the T-even baseplate with the fold-out STFs fixing the post-infection baseplate at about 20 nm distance from the outer membrane—the mechanical force of the STF deployment may serve to drive the receptor-recognizing domains of these fibers through the protective OPS layer. We also demonstrate that direct O antigen recognition by LTFs is not the only strategy available for T-even-like viruses to gain a wide host range. Brandy49 phage, despite its close relatedness to other viruses studied by us, clearly uses another (and very effective) mechanisms, the molecular detail of which remain to be elucidated.

## Figures and Tables

**Table 1:** Plaque formation by RB49-like bacteriophages on different *E. coli* strains.

Phages	Host Strain (O Antigen Type)							
	4s (O22)	4sR (Rough)	F17 (New)	F17 WbbL (Rough)	F5 (O28)	F5:24B (Rough)	MG1665 K-12 (Rough)	C600 (Rough)
RB49	−	−	−	−	+	+	+	+
RB49-EK	−	−	+	−	+	+	+	+
Whisky49	−	−	+	−	+	+/−	+/−	+/−
Cognac49	−	−	−	−	+	+	+	+
Brandy49	+	+	+	+	+	+	+	+

*E. coli* “wild” strains are highlighted in bold to distinguish them from laboratory-modified derivatives. “+”—EOP > 0.1; “+/−”—10^−2^ < EOP < 10^−1^; “+/−−”—10^−3^ < EOP < 10^−2^; “−”—no plaques observed.

**Table 2:** Plaque formation on *E. coli* strains, selected for phage resistance.

Host Strains	Bacteriophages					
	Cognac49	Whisky49	RB49	RB49-EK	Brandy49	FimX
F5 wt	+	+	+	+	+	− (EOP~10^−6^)
F5-rCognac49	−	−	−	−	+	+
F5-rWhisky49	−	−	−	−	+	+
F5-rRB49	−	−	−	−	+	+
F5-rRB49-EK	−	−	−	−	+	+
F5-rRB49:pOmpA	−	−	−	−	+	+
F17 wt	−	+	−	+	+	−
F17-rWhisky49	−	−	−	−	+	+
F17-rRB49-EK	−	−	−	−	+	+
F17-wbbL:pOmpA	−	−	−	−	+	+
K-12 MG1665	+	+/−	+	+	+	+
K-12 ΔompA	−	−	−	−	+	+
K-12 ΔompA:pOmpA	+	+/−	+	+	+	+
K-12-rRB49	−	−	−	−	+	+
K-12-rRB49:pOmpA	−	−	−	−	+	+
